# Enhancement of Phytosterol and Triterpenoid Production in Plant Hairy Root Cultures—Simultaneous Stimulation or Competition?

**DOI:** 10.3390/plants10102028

**Published:** 2021-09-27

**Authors:** Agata Rogowska, Anna Szakiel

**Affiliations:** Department of Plant Biochemistry, Institute of Biochemistry, Faculty of Biology, University of Warsaw, 1 Miecznikowa Street, 02-096 Warsaw, Poland; a.rogowska@biol.uw.edu.pl

**Keywords:** hairy roots, plant in vitro cultures, saponins, squalene cyclization, sterols, triterpenoids

## Abstract

Plant in vitro cultures, including hairy roots, can be applied for controlled production of valuable natural products, such as triterpenoids and sterols. These compounds originate from the common precursor squalene. Sterols and triterpenoids distinctly differ in their functions, and the 2,3-oxidosqualene cyclization step is often regarded as a branch point between primary and secondary (more aptly: general and specialized) metabolism. Considering the crucial role of phytosterols as membrane constituents, it has been postulated that unconstrained biosynthesis of triterpenoids can occur when sterol formation is already satisfied, and these compounds are no longer needed for cell growth and division. This hypothesis seems to follow directly the growth-defense trade-off plant dilemma. In this review, we present some examples illustrating the specific interplay between the two divergent pathways for sterol and triterpenoid biosynthesis appearing in root cultures. These studies were significant for revealing the steps of the biosynthetic pathway, understanding the role of particular enzymes, and discovering the possibility of gene regulation. Currently, hairy roots of many plant species can be considered not only as an efficient tool for production of phytochemicals, but also as suitable experimental models for investigations on regulatory mechanisms of plant metabolism.

## 1. Introduction

Plant-derived bioactive constituents are highly valuable in numerous contemporary pharmaceutical and industrial applications. Synthesis and accumulation of these compounds are usually limited to particular plant species, their developmental stage or in special conditions related to stress. The valuable phytochemicals are often found in native plants in extremely low abundance for profitable extraction. Moreover, traditional extraction methods have high associated costs and generates various by-products that are harmful to the environment. The economical demand for native plant products causes several problems: the risk of extinction from over-exploitation of certain species, difficulties in domestication and further cultivation of wild medicinal plants, and geopolitical problems triggered by exploitation of exotic plant resources [1]. Hence in vitro cultures are advantageous because of their well-defined, controlled, and reproducible growth conditions without the limitations of natural factors such as climatic variations, geographical location, plant age, and vegetative phases [2,3,4]. Plant in vitro cultures offer condensed biosynthetic cycles with short cultivation time, in addition to the possibility of adjusting the optimal conditions required for production of the bioactive constituents [5]. Additionally, they have a high potential for genetic engineering because they do not interact with the environment in the same manner as genetically modified crops do, so they are regarded as safer and are readily accepted [6]. Also, the isolation of synthesized phytochemicals can be more rapid and efficient than in the case of intact whole plants [7]. There are many types of plant in vitro cultures used in biotechnology for boosting yields of valuable compounds, from cell suspension cultures to organized structures of plant organs, like roots [8]. However, the cultured undifferentiated cell suspensions often fail to synthesize and accumulate the desired metabolites because of their instability, non-uniformity of the product formation, and sometimes simply the lack of storage sites. Specialized metabolism and tissue-specific localization in plants are under strict regulation, therefore the development of a certain level of differentiation is crucial for the successful biosynthesis of many phytochemicals. The hairy root cultures are particularly advantageous in that respect. Additionally, hairy root cultures have high growth rate without an external supply of phytohormones, low doubling time, genetic and biochemical stability, ease of maintenance, and the ability to synthesize a wide range of phytochemicals [9,10,11].

## 2. Hairy Root Cultures

The term ‘hairy root’ was used in the scientific literature for the first time in 1900 to describe a special disease of fruit crops, occurring in vineyards, plum, apple, and peach nurseries [9]. This disease was later reported in 1934 as ‘hairy root syndrome’ for distinctive formation of small, thin hair-like mass of roots appearing as a result of *Agrobacterium rhizogenes* infection [11,12]. At present, this Gram-negative, rod-shaped, pathogenic soil bacterium, also known as *Rhizobium rhizogenes*, is one of the best-known representatives of genus *Agrobacterium* of family *Rhizobiaceae*. The mechanism of *Agrobacterium*-mediated natural transformation has been known and applied in biotechnology for more than 30 years [13].

Four major sequential events occur during this gene transfer phenomenon: (i) chemotaxis, where phenolic exudates (e.g., acetosyringone) from the wounded plant stimulate attachment of the bacteria to explant/root cells; (ii) generation of single-strand T-DNA in the bacterial plasmid (called Ri for root-inducing) and its translocation to plant host genome; (iii) Ri plasmid T-DNA incorporation and expression in the host genome; (iv) formation of either tumors or hairy roots at the infection sites of the host plant, depending on the type of plasmid in the bacterial cell [2,13,14]. Successful gene transfer requires the involvement and adequate coordination of both, bacterial and host factors, to complete the transformation and produce hairy roots phenotype.

The T-DNA contains two independent regions, TL (left T-DNA) and TR (right T-DNA), both are transferred and integrated independently into the host plant genome. However, only TL-DNA is crucial and sufficient for hairy roots induction. Sequence analysis of TL-DNA has revealed four open reading frames (*rolA*, *rolB*, *rolC*, and *rolD*). The rol genes products play key role in hairy roots formation; however, the *rolB* gene seems to be the most important in hairy root induction. It was also discovered that knock-out of the *rolB* gene causes a virulence of the plasmid [15]. So far the complete mechanism of action of these genes is unknown. Nevertheless, there are many interesting aspects of interactions between the *rol* genes and the plant genes, and factors that may lead to alterations in plants sensitivity, hormones concentrations and plant secondary metabolism [16].

The natural plant hosts of *A. rhizogenes* are not numerous and they belong only to a few dicotyledonous species. However, under laboratory conditions, hairy roots can also be induced in various monocotyledonous and gymnosperm plants. Thus, currently, more than 400 plant species belonging to 50 families of angiosperm plants representing 150 genera are exploited for their hairy roots [10,11]. Meanwhile, the technique of infecting the plants with *A. rhizogenes* offers a promising system for various manipulations required for specialized metabolite production and owing to it *A. rhizogenes* is also called a “natural genetic engineer” [9,17].

Hairy root cultures (HRCs) can accumulate valuable phytochemicals to the levels comparable to that of an intact plant and they are usually stable in their biosynthetic capacity [18]. Indeed, stimulation of specialized plant metabolism (regarded as a mechanism involved in defense responses in wild plants) is a well-known phenomenon in HRCs, often explained by the activating effect of root-inducing genes (*rol*-genes) [9]. The reported extent of specialized metabolism activation varied from 2- to 300-folds depending on the plant species and type of metabolite. The mechanism of stimulation of specialized plant metabolism by *rol*-genes is still not clear. It is believed that *rol*-genes cause perturbation in plant’s stress responses and defense strategies resulting in uncommon signal transduction pathways in plants. It was shown that *rol*-genes act independently of typical plant defense mechanisms involving hormones (ethylene, jasmonic and salicylic acid) and the calcium-dependent NADPH-oxidase pathways [9,19].

HRCs can also produce phytochemicals synthesized in roots of the native plant. Interestingly, they can also accumulate metabolites that are normally exclusively found in the aerial parts of an intact plant [3] and can also synthesize new types of compounds, originally not found in the native plants [9,20]. Although it is claimed that hairy roots can synthesize stable amounts of phytochemicals, the accumulation of the desired compounds can be limited due to the feedback inhibition loops if they are poorly released into the medium [18,21].

**Figure 1:**
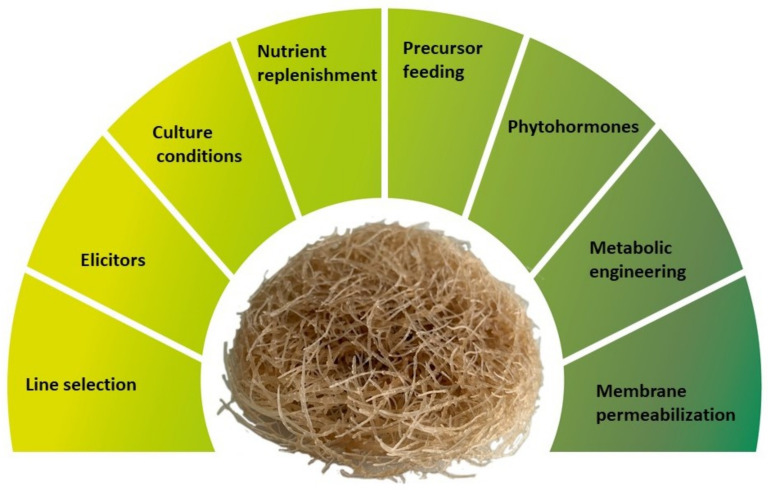
The main strategies applied to improve productivity of hairy root cultures.

To ensure the economic viability of production of phytochemicals by HRCs or other plant in vitro cultures it is crucial but difficult to achieve high yields of biosynthesis and extraction along with market prices that can compete with traditional production processes (traditional cultivation or chemical synthesis). The lack of commercial success generally results from very low productivity of obtained cultures and difficulties to scale up the production. Several biotechnological strategies have been developed to improve HRCs productivity (Figure 1). This includes, among others, screening and selection of high yield lines, application of elicitors, and optimization of culture media and culture conditions (i.e., optimal levels of salt, sugar, nitrogen, phosphate, and physical factors such as temperature, illumination, light quality, medium pH, agitation, aeration) [22,23,24,25]. Other strategies for improving HRCs productivity include replenishment of nutrients and precursor feeding, in situ product removal to overcome feedback inhibition, application of phytohormones in medium and metabolic engineering, permeabilization of membranes to improve metabolite secretion, and scale-up to bioreactors [22,26]. To achieve the best results, some of these methods can be combined in one treatment to generate the synergistic effect, e.g., elicitation and precursor feeding [5].

## 3. Elicitation

**Figure 2:**
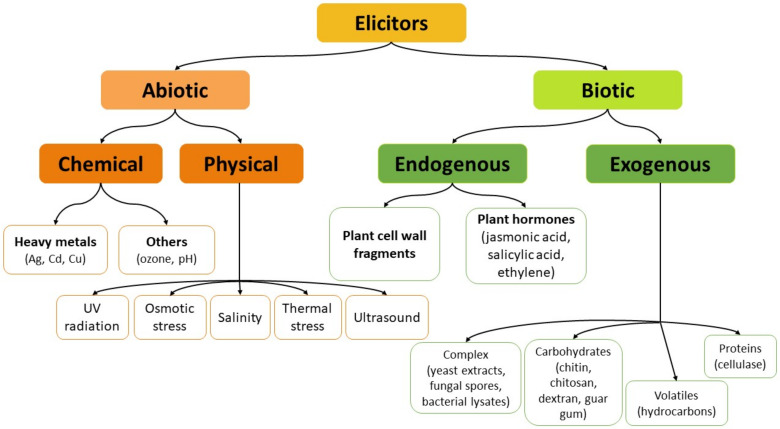
The classification of elicitors into abiotic and biotic factors.

Elicitation is one of the most practically feasible and most effective strategies for enhancing the production of specialized metabolites in plant biotechnology. For intact plants, elicitation can be considered as a defense response towards stress conditions or factors, resulting usually in a process of inducing or enhancing the synthesis of plant defense metabolites to ensure plant survival, persistence, and competitiveness [27]. In biotechnology, elicitation is often defined as the induced or enhanced biosynthesis of metabolites due to the addition of trace amounts of elicitors [8,28]. On the basis of their characteristics, elicitors can be divided into physical or chemical; on the basis of their origin they can be divided into exogenous (pathogen origin) and endogenous (compounds released from plants after infection by a pathogen); on the basis of the plant’s response to them, they can be divided into specific and general. However, the most convenient and widely accepted is the classification into two categories: abiotic and biotic (Figure 2) [29,30].

Abiotic elicitors can be categorized as either chemical substances of non-biological origin, e.g., mineral salts, heavy metals, or physical factors/conditions, like light (UV-B, UV-C radiation), temperature (heat or cold), ultrasound, osmotic stress. Biotic elicitors include substances of external, pathogenic origin (exogenous factors) and compounds synthesized by plants after the pathogen action (endogenous factors) [30]. Exogenous elicitors are usually released by microorganisms and other pathogens. This category embraces fungal or bacterial lysates, yeast extracts, microbial enzymes, polysaccharides from cell walls (chitin, chitosan, glucans), and even entire fungal spores. Endogenous elicitors include polysaccharides released from the plant cell walls as a result of the pathogen attack, some intracellular proteins, as well as effectors synthesized in response to different types of stress, like methyl jasmonate and salicylic acid. The last two effectors are also defined as plant growth regulators or phytohormones because they induce cellular responses at low concentrations, distant from the site of synthesis [28,31]. From the chemical point of view, biotic elicitors can be characterized as factors of defined composition (e.g., carbohydrates—pectin, chitosan, oligosaccharides; proteins—cellulase), or of complex and sometimes unknown composition (e.g., yeast cell wall, mycelia cell wall, fungal spores) [30].

Intensive research effort has been devoted to investigating the molecular mechanisms of elicitation, particularly the biochemical responses induced by biotic elicitors. A plant’s response to elicitor-induced stress mostly begins at the cell plasma membrane, since the elicitor binds to a plasma membrane receptor, leading to the action of secondary messengers, which amplify the signal for various downstream reactions. There are sequentially occurring events in defense responses induced by elicitors, which include reversible phosphorylation and dephosphorylation of the membrane and cytosolic proteins, changes in ion fluxes across the membrane (i.e., enhancement of cytosolic Ca^2+^), rapid changes in protein phosphorylation patterns and protein kinase activation, stimulation of mitogen-activated protein kinase (MAPK), and activation of G-protein. NADPH oxidase is activated, which results in the production of reactive oxygen and nitrogen species (ROS and RNS). Synthesis of other secondary messengers (like inositol 1,4,5,-trisphosphate and diacyl glycerol) takes place, mediating nitric oxide and the octadecanoid signaling pathway. Early defense gene expression leads to the octadecanoid pathway, in which linolenic acid is converted to jasmonic acid; subsequently jasmonates are synthesized as secondary messengers. In the next step, the late defense genes are transcriptionally activated, which results in the production, distribution, and accumulation of specialized metabolites. Plants’ systemic responses lead to the production and accumulation of antimicrobial compounds such as phytoalexins and pathogenesis-related proteins, which play a key role in defense against pathogens. Cytoskeleton undergoes general reorganization and there may occur structural changes in the cell wall like lignin deposition to reinforce these structures. Thus, elicitor signal transduction is a network composed of multiple components consisting of parallel or cross-linking signaling pathways leading to different target responses. Numerous studies show that two or more signaling pathways are involved in the regulation of the defensive cellular process. In such cases, crosstalk among multiple signaling pathways is a prominent mechanism in plant signal transduction networks that enables plants to modulate different sets of genes temporally and spatially in a range of situations against many types of stress [8,32,33,34].

## 4. Competition between General and Specialized Metabolism in Plant Response to Elicitation

It can be expected that external abiotic conditions (such as light, temperature, UV irradiation, soil fertility, salinity, the presence of ions of heavy metals), as well as biotic factors (such as the attack of herbivores, pathogen infection, allelopathic influence of neighboring plants) might affect processes associated with plant growth and development, leading to significant alterations in the primary (general) metabolism. The synthesis of specialized secondary metabolites plays a strategic role in plant response to stress and elicitation but, as a consequence, the increased production of bioactive substances results due to redirection of the metabolic resources to the specialized metabolism at the expense of growth and basic physiological functions [35] This is a classical “Cornelian dilemma” of plants—to grow or to defend themselves [36,37]. Because primary and secondary metabolites generally have the same chemical precursors (substrates, cofactors), a classical explanation of the growth-defense trade-off is that a global pool of resources has to be allocated to one mechanism or the other, so finally the high metabolic cost of one process may indirectly impact the other. If priority is given to the plant growth processes, the availability of these resources may become limiting for plant defense-related processes, and vice versa. However, more and more studies challenge this resource-based hypothesis, pointing that many primary and secondary metabolites are multifunctional and serve auxiliary roles. The separation of plant metabolism into primary and secondary is an oversimplified view and does not adequately describe the complexity of processes that plants have evolved to grow, develop and simultaneously cope with a myriad of abiotic and biotic stresses [37,38].

The plant “Cornelian dilemma” acting in the natural environment can be regarded as potential challenges in the new “eco-friendly” methods of sustainable agriculture which are based on the induction of the plant immunity with the use of elicitors and can lead to reduced plant growth and vigor. However, it is not clear how to interpret the relations between so-called general and specialized metabolism in plant in vitro cultures, where plant cells are maintained in the medium containing all required nutrients, and no stress factors (or at least not critically dangerous for survival) are acting. In many cases, the lack of the production of specialized metabolites in plant in vitro cultures is regarded as a consequence of the absence of stress, leading to suppression of the biosynthesis of defense metabolites. Hence the need for elicitation and other techniques, which were discussed in the previous chapters. However, the problem is deeper and more complex. Some specialized compounds are synthesized in plant in vitro cultures without elicitation. Some elicitors provoke the synthesis of the compounds not produced by the native plants [20]. Specialized metabolites have numerous applications for humans in many branches of the industry but their biological roles in plants remains elusive, and proposed functions lack an identified underlying molecular mechanism. Understanding the function of specialized metabolites can be hampered by their specific spatiotemporal biosynthesis and accumulation within plant tissues and organs throughout the cycle of plant growth and development [39]. One such group of compounds embracing a variety of compounds with diverse but not always clearly defined functions, classified either as general or specialized metabolites and thus potentially competing, are terpenoids. Their production in plant in vitro cultures, including hairy roots, is an important field of investigation in biotechnology.

## 5. Biosynthesis of Terpenoids

Terpenoids, also known as isoprenoids, are a group of structurally diverse phytochemicals associated with general (primary) as well as specialized (secondary) metabolism. Some isoprenoids play function as phytohormones (abscisic acid, cytokinins, gibberellins, brassinosteroids), some are the components of electron carriers (cytochrome a, quinones), some are directly indispensable for photosynthesis (carotenoids, the chain of phytol in chlorophylls) or for membrane permeability and fluidity (sterols). Thus, numerous isoprenoids are involved in basic plant functions and participate in the regulation of processes of growth and development. The other compounds of this group have less recognized functions and therefore they have been classified as specialized metabolites. However, they are considered crucial for various mechanisms of plant chemical defense and their interaction with the environment. They often serve as pollinator attractants or herbivore repellents, antibiotics, or toxins [40,41].

According to the number of carbon atoms, terpenoids are classified as hemi-, mono-, sesqui-, di-, ses-, tri-, tetra- and polyterpenoids. This irregularity comes from their biosynthetic pathway involving the repetitive fusion of “isoprene units” (C_5_H_8_). In higher plants, the biosynthesis begins with the formation of the principal precursor, isopentenyl diphosphate (IPP), which can be derived from two parallel pathways: cytoplasmic mevalonate (MVA)/3-hydroxy-3-methyl-glutaryl-CoA reductase (HMGR) pathway or plastidial 2-*C*-methyl-D-erythritol 4-phosphate (MEP) pathway, also known as 1-deoxy-D-xylulose 5-phosphate (DOXP)/ non-MVA pathway. The consecutive condensations of IPP and its allylic isomer, dimethylallyl diphosphate (DMAPP) leads to the formation of the precursors of the different terpenoid classes. Afterwards, the arrangement of various skeletons and their modifications occur creating the structural and functional multiplicity of isoprenoids. Three types of chemical reactions form subsequent classes of terpenoids: elongation, condensation, and cyclization. The multilayered regulatory network leads to the production of one of the most diverse groups of molecules in plants [41,42,43,44].

Plants are unique in maintaining both isoprenoid biosynthetic pathways parallelly, whereas most other organisms (with few exceptions as *Streptomyces*) use only one of them. The existence of two alternative and spatially separated pathways using different pools of substrates is believed to be beneficial for plants as sessile organisms interacting with rapid demand for defense compounds to respond to their environment under the influence of various abiotic and biotic stresses. The possibility of metabolic crosstalk (and thus cross-flow of intermediates) between these two pathways exists since the compounds with mixed origin were proved to be synthesized, particularly when one of these pathways is temporarily blocked or down-regulated. Typically, the biosynthesis of carotenoids and phytyl chains of chlorophyll takes place in plastids by MEP pathway, that of triterpenoids—in cytosol by MVA pathway, whereas some monoterpenes, sesquiterpenes, and polyterpenes can be formed from units of both origins, thus delivering “mosaic” structure [43,45].

## 6. The Problem of the Common Pathway in Triterpenoid Biosynthesis

Triterpenoids represent a large group of plant isoprenoids synthesized from the C30 precursor: squalene [42,46]. Squalene is formed as a result of two subsequent condensations: “head-to-tail” condensation of two IPP units with a DMAPP unit leading to C15 farnesyl diphosphate (FPP), and successive “head-to-head” fusion of two FPP units. Squalene is a linear hydrocarbon, which afterward is oxidized to 2,3-oxidosqualene and then cyclized by special enzymes, oxidosqualene cyclases, into various tetra- or pentacyclic structures [41].

**Figure 3:**
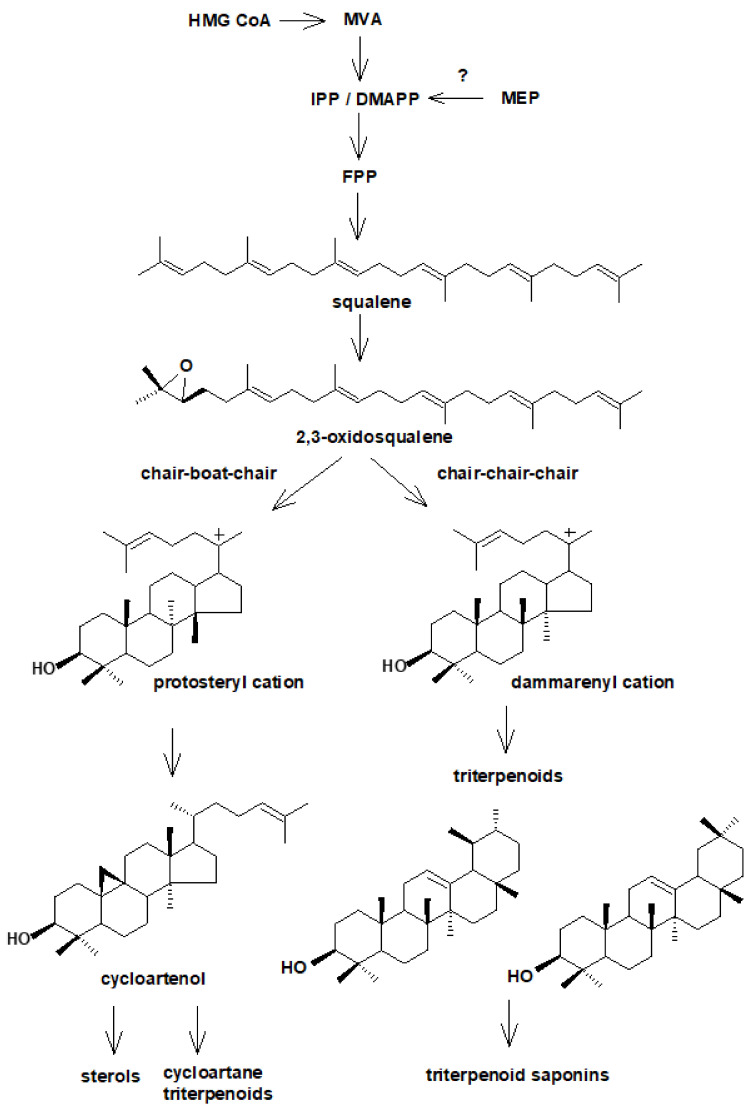
The simplified scheme of divergent biosynthetic pathways of sterols and triterpenoids.

The cyclization of 2,3-oxidosqualene represents one of the most sophisticated and versatile chemical reactions found in nature [47]. Oxidosqualene cyclases (OSCs) catalyze a series of cation-π cyclizations and 1,2-rearrangement reactions of a linear substrate to produce a wide range of polycyclic products. Thus, the structural diversity of squalene-derived compounds arise mainly from OSC reactions that precisely control the conformation of the substrate, the geometry of cyclization patterns, and the fate of transient carbocation intermediates. Consequently, according to the mode of formation, the compounds originating from squalene cyclization can be divided into steroids, i.e., tetracyclic compounds based on perhydro-1,2-cyclopentano-phenantren moiety, and triterpenoids possessing usually 4- or 5-ring carbon skeleton of various arrangements. The decisive step is, therefore, a conformation of oxidosqualene during the cyclization of the first three rings: *chair-chair-chair* conformation leading to the formation of the majority of triterpenoids (such as β-amyrin, α-amyrin, and lupeol that are found in plants), and *chair-boat-chair* conformation leading to the formation of the protosteryl cation that generally gives rise to sterol precursors, cycloartenol and lanosterol, although some triterpene precursors such as parkeol and cucurbitadienol can also be produced [41,47,48,49]. Cycloartenol, which is considered to be a general precursor of sterols and steroid hormones in plants, can be also classified as pentacyclic triterpene, although it is based on tetracyclic lanostane structure. In some plants, cycloartenol gives rise to cycloartane triterpenoids (Figure 3) [50]. Some studies on plant genome suggested that there are enzymes enable to convert 2,3-oxidosqualene to lanosterol, what undermines concept that cycloartenol is the only precursor for sterol biosynthesis in plants [49].

Plant sterols can be classified either as 4-desmethylsterols, 4-methylsterols, or 4,4′-dimethylsterols. The most abundant are 4-desmethylsterols, e.g., sitosterol, campesterol, and stigmasterol. However, over 200 different sterol structures occurring in various plant species have been reported. The most common phytosterols have a double bond at position 5 of the B-ring and they are commonly referred to as Δ^5^. Some others have a double bond at position 7, they are named Δ^7^-phytosterols. Plant sterols can exist in plants in a free form, as esters with fatty or phenolic acids, or as glycosides and acylated steryl glycosides [51,52].

Triterpenoids can be classified according to the type of their skeleton into dammaranes, oleananes, ursanes, lupanes, taraxasteranes etc. They can exist in plants in free or bound forms, i.e., as esters or glycosides, referred to as saponins. Sterols and triterpenoids distinctly differ in their functions, and therefore they are commonly regarded as general and specialized metabolites, respectively. Sterols are constituents of plant membranes and they participate in the regulation of their fluidity and permeability, they also serve as precursors of brassinosteroid hormones, whereas triterpenoids are believed to play an important role in plant chemical defense and interactions with the environment. Therefore, the step of 2,3-oxidosqualene cyclization is often regarded as a branch point between primary and secondary (more aptly: general and specialized) triterpenoid metabolism, however, saponins containing steroidal sapogenins are considered specialized compounds, as triterpenoid saponins [41].

Since both sterols and triterpenoids are synthesized as products of one common precursor, 2,3-oxidosqualene, the trials of stimulation of triterpenoid biosynthesis in plants and plant in vitro cultures including hairy roots often concern the problem of the possible competition. Already in early experiments on triterpenoid production in plant in vitro cultures, it was observed that the intensity of the biosynthesis of pentacyclic triterpenoids was dependent on the growth phase of the culture in a characteristic manner, i.e., it decreased after callus inoculation and initiation of suspension culture and then increased rapidly during the middle and late exponential phases of growth. Simultaneously, the sterol content remained rather constant from the exponential and early stationary phase until the late stationary phase. The experiment was performed on suspension cultures of three plant species: *Datura innoxia* (Solanaceae) producing betulinic acid, *Luffa cylindrical* (Cucurbitaceae) producing bryonolic acid, and *Lycopersicon esculentum* (Solanaceae) producing lupeol. Considering the crucial role of phytosterols as membrane constituents, it has been postulated that unconstrained biosynthesis of triterpenoids can occur when sterol formation is already satisfied, and these compounds are no longer needed for cell growth and division [53,54]. This hypothesis seems to follow directly the growth-defense trade-off plant dilemma, however, the results obtained in various other experiments on the plant in vitro cultures were more ambiguous and the observed phenomenon deserves further careful consideration.

In elicitation experiments performed on cell suspension cultures of *Uncaria tomentosa* (Wild.) DC, commonly known as cat’s claw, the content of triterpenoids (ursolic, oleanolic and quinovic acids) increased significantly (up to 16-fold) after treatment with pectin, and less markedly (approx. 2–3 times) after treatment with fungal elicitors, whereas neither growth of the culture nor sterol accumulation was affected [54]. However, the symptoms of competition between triterpenoid and sterol pathways appeared more clearly after incubation of these cultures with [5-^3^H] mevalonic acid, wherein the control (not elicited) cultures the incorporation of the radioactive labeled precursor was higher in phytosterols (20%) than pentacyclic triterpenoids (14%), whereas elicited cell cultures incorporated more radioactive labelled precursors into triterpenoids (23%) than sterols (15%). Thus, elicitation seems to influence the channeling of carbon flux into the triterpenoid branch of the pathway.

Another well-documented example of this competition between sterol and triterpenoid biosynthesis pathways was the report on cell suspension cultures of *Tubernaemontana divaricata* (L.) R. Br. ex Ruem. et Schult. [55]. This plant is a rich source of bioactive monoterpene indole alkaloids (e.g., vallesamine, *O*-acylvallesamine, vaophylline). Cell suspension cultures were treated with *Candida albicans* elicitor preparation to improve alkaloid production, instead, the increase of triterpenoids (not found in control cultures) was observed accompanied by a significant decrease (by 50%) in culture mass growth. Afterwards, in feeding experiments with labeled precursors, [1-^14^C]IPP, [1-^3^H_2_]FPP i [2-^14^C]MVA, it was demonstrated that in elicited cultures the radioactivity was incorporated mainly in triterpenoids (squalene, squalene-2,3-oxide, α- and β-amyrins, oleanolic and ursolic aldehydes and acids), whereas unelicited cells synthesized squalene and small amounts of cycloartenol only. Experiments with [2-^14^C]MVA indicated that in elicited cultures the synthesis of phytosterols (and hence the culture growth) was inhibited at the level of squalene 2,3-oxide: cycloartenol cyclase.

Similarly, when the first trials of obtaining triterpenoid saponin glycyrrhizin (diglucoside of glycyrrhizic acid) from in vitro cultures of *Glycyrrhiza glabra* were not successful, the tracer (labeling) method with the use of precursors: [1-^14^C]acetate and [2-^14^C]MVA was applied to investigate the respective pathways in the plant organs and in the in vitro cultures [56]. Intensive labeling of sterol precursors, i.e., cycloartenol and 24-methylenecycloartanol, and practically no labeling of β-amyrin (precursor of glycyrrhitic acid) was observed in callus cultures. In turn, the simultaneous labeling of cycloartenol and β-amyrin was detected in plant organs and adventitious root culture, leading to the conclusion that triterpenoid biosynthesis, in contrast to sterol formation, requires a certain level of differentiation.

During the last decades, triterpenoid and saponin production in plant in vitro cultures has been the subject of many studies and numerous reports. In this review, we present only some examples illustrating the specific interplay between the two divergent pathways for sterol and triterpene biosynthesis appearing in hairy and adventitious root cultures.

## 7. Sterols versus Triterpenoids in Hairy Roots—Competition

One of the most studied plants regarding biotechnological attempts to stimulate the biosynthesis of triterpenoids is *Panax ginseng*. This plant has been used as an herbal medicine in East Asia for thousands of years and holds an important position among traditional medical and health-supporting products worldwide. Characteristic saponins synthesized by *P. ginseng*, referred to as ginsenosides, are classified based on their aglycone structures: dammarane-type (tetracyclic) and oleanane-type (pentacyclic). The major ginsenosides are of the dammarane-type, which can be further categorized into protopanaxadiol and protopanaxatriol subspecies [57]. Thus, it was previously recognized that oxidosqualene cyclization by β-amyrin synthase (bAS), dammarenediol synthase (DS), and cycloartenol synthase (CS) constitute the branching point for triterpenoid and sterol biosynthesis in ginseng. As ginsenosides and phytosterols share the same precursor, 2,3-oxidosqualene, it is likely that the suppression of CS, which is responsible for phytosterol synthesis, ultimately would lead to an increased number of precursors available for ginsenoside biosynthesis. This suppression of CS was demonstrated by antisense expression of a CS fragment in transgenic ginseng hairy roots [56]. Phytosterol levels from antisense transgenic lines decreased by roughly 50%, while total ginsenoside content was 50–100% higher compared to control roots. However, antisense-CS manipulation had deleterious effects on hairy root growth particularly during early days of the culture, indicating a correlation between low phytosterol levels with poor initial growth of hairy roots [58].

The possibility of the stimulation of biosynthesis of phytosterols and saikosaponins (pentacyclic oleanane-type triterpenoid saponins) was investigated in experiments performed on wild-type and transgenic adventitious roots of medicinal plant *Bupleurum falcatum* L. [59]. The relation between sterol and triterpenoid pathways was different in obtained transgenic constructs overexpressing *B. falcatum* squalene synthase (*BfSS1*) gene in the sense and antisense orientations. The content of total phytosterols, including sitosterol, campesterol, and stigmasterol, markedly increased in sense transgenic roots but decreased in antisense transgenic roots as compared to wild-type roots. After elicitation with methyl jasmonate (MeJA), saikosaponin production was strongly stimulated in wild-type roots, whereas simultaneously the phytosterol production was suppressed. A similar effect of suppressed phytosterol accumulation was observed in antisense transgenic roots, in contrast to transgenic roots overexpressing *BfSS1* in the sense orientation, which resulted in enhanced production of both phytosterols and saikosaponins. Thus, this experiment revealed the antagonistic interaction between sterol and triterpenoid pathways occurring after MeJA elicitation in wild-type roots of *B. falcatum*, which could be abolished in transgenic roots overexpressing *BfSS1* in the sense orientation [59].

The effects of overexpressing various genes involved in triterpenoid biosynthesis are not easy to predict, and obtained results are sometimes unexpected. The hairy roots of *Centella asiatica* (L.) Urban were transformed with a construct of ginseng farnesyl diphosphate synthase (*PgFPS*) to stimulate the production of triterpenoid saponins [60]. *C. asiatica* is a medical herb containing a variety of triterpenoid saponins (mainly oleanane- and ursane-type, including asiaticosides, madecassocides, and centellasaponins). The obtained lines of *C. asiatica* hairy root overexpressing *PgFPS* accumulated increased levels of squalene, one of the transgenic lines exerted the 3-fold higher content of sterols (cholesterol, campesterol, sitosterol, and stigmasterol), whereas no increase either in free triterpenoids (α-amyrin, β-amyrin, and lupeol) or saponins was detected. The synthesis of madecassoside and asiaticoside in transgenic hairy roots was only slightly (1.15-fold) increased after 14 days of treatment with MeJA, but decreased after 28 days of elicitation, suggesting that overexpression of farnesyl diphosphate synthase finally resulted in feed-back suppression of downstream steps of the pathway [60]. Unfortunately, in this experiment, the content of sterols in hairy roots after elicitation was not determined.

The study on elicitation of hairy roots derived from medicinal plant rich in olenanane-type saponins, marigold *Calendula officinalis* [61] showed that treatment with jasmonic acid resulted in a significant increase of saponin accumulation in hairy root tissue (up to 20-fold) and their release to the medium (up to 113-fold), however, simultaneously a very sharp decrease in the content of sterols (approx. 60%) and the reduction in hairy root mass was observed. Moreover, jasmonic acid (JA) elicitation changed the quantitative profile of phytosterols, resulting in a 4-fold decrease in isofucosterol and campesterol content in elicited roots, 2-fold decrease in sitosterol content, accompanied by approx. 2-fold increase in cholesterol and sitostanol levels. These results suggested that the metabolic response of *C. officinalis* hairy roots to elicitation with jasmonic acid involved redirection of the carbon flow between the two competing pathways, and after JA treatment, favored the biosynthesis of defense compounds over metabolites involved in basic metabolism [61]. Hairy roots cultures do not require any external supply of phytohormones to the medium for their normal growth, however, the addition of growth regulators like auxins and cytokinins, which generally affect plant defense responses, can lead to metabolic modifications. The addition of synthetic cytokinin, 6-benzyl- aminopurine (BAP), resulted in a 32% increase in free oleanolic acid content in hairy roots tissue of *C. officinalis*, over 10-fold enhancement of release of oleanane saponins to the medium, simultaneously causing a decrease in the total sterol content (by 17%). These results suggest that some plant growth regulators like BAP may have a similar effect on sterol and triterpenoid metabolism as jasmonic acid [62].

## 8. Sterols and Triterpenoids in Hairy Roots—Parallel Enhancement

Genetic modifications seem to be an effective solution to address the competition between sterol and triterpenoid biosynthesis among plant in vitro cultures. *P. ginseng* contains squalene synthase and dammarenediol synthase which are involved in the biosynthesis of squalene and dammarane-type ginsenosides, and have been used to generate transgenic ginseng models [63]. For example, overexpression of the squalene synthase *PgSS1* in transgenic *P. ginseng* adventitious root cultures resulted in enhanced production of phytosterols and triterpenoids. Sterol content (campesterol, sitosterol, stigmasterol) increased 2-fold, whereas the saponin levels induced 1.6- to 3-fold compared to wild-type. However, in this study it was observed that MeJA elicitation of untransformed *P. ginseng* adventitious roots resulted in increased squalene synthase *PgSS1* mRNA, accompanied by activation of squalene epoxidase (*SE*), *β*-amyrin synthase (*bAS*), but not cycloartenol synthase (*CAS*) [63]. Thus, the competition between sterol and triterpenoid synthesis did not occur in genetically modified adventitious roots, but still appeared in untransformed roots after elicitation.

An analysis on the early and middle steps of the triterpenoid biosynthesis pathway, i.e., the formation of isoprenyl diphosphate (IPP) by mevalonate-5-pyrophosphate decarboxylase (MVD), as well as the formation of farnesyl diphosphate (FPP) derived from IPP and dimethylallyl diphosphate by farnesyl pyrophosphate synthase (FPS), was conducted as well [64]. The genes governing the expression of these enzymes, *PgMVD* and *PgFPS*, were transformed into *P. ginseng* hairy roots. *PgMVD*- and *PgFPS*-overexpressing lines showed varied intensity of sterol and triterpenoid biosynthesis. Concerning *PgMVD* overexpression, sterol concentrations (including campesterol, stigmasterol, and β-sitosterol) increased up to 4.4-fold higher than the control, whereas the levels of ginsenoside and β-amyrin decreased. In contrast, both ginsenoside and sterol contents were enhanced in the *PgFPS*-transgenic lines from 2.4-4.6-fold, respectively. These data suggest that *PgFPS* overexpression may upregulate cycloartenol synthase and dammarenediol synthase, resulting in stimulation of biosynthesis of sterols and ginsenosides. In contrast, *PgMVD*-overexpression likely only regulates cycloartenol synthase. It has previously been postulated that the amount of any compound that can be produced by the engineering of a single gene is limited, and the best solution is to use the current multigene transformation methods to increase the production of specialized metabolites [64].

Another study on genetic transformation which led to metabolic engineering of the sterol and triterpenoid pathway was performed on *Platycodon grandiflorum* A. DC. [65]. *P. grandiflorum* is a medicinal plant involved in platycodin synthesis, a group of oleanane-type triterpenoid saponins. *P. grandiflorum* hairy roots were transformed with a construct expressing *Panax ginseng* 3-hydroxy-3-methylglutaryl-coenzyme A reductase (PgHMGR), an enzyme which catalyzes the rate-limiting step of the mevalonate pathway. This transformation resulted in increased production of phytosterols and triterpenoids. In transgenic hairy root lines, total platycoside and phytosterol (α-spinasterol) contents increased by 1.5–2.5-fold and 1.1–1.6-fold, respectively. While the content of platycosides and α-spinasterol increased upon *PgHMGR* overexpression, β-amyrin was slightly decreased. These data can be explained by enhanced expression of hydroxylases and sugar transferases involved in saponin biosynthesis, which results in minimal levels of free aglycon [65].

Sometimes genetic modifications are not necessary to attenuate competition between sterols and triterpenoids, and similar effects can be obtained after treatment with certain elicitors. This situation was observed after elicitation of *C. officinalis* hairy roots with chitosan [61], which resulted in a 2-fold increase in accumulation of oleanolic acid saponins in hairy root tissue, a 3-fold enhancement of their release to the culture medium, and a slight induction (18%) in sterol biosynthesis. Additionally, chitosan treatment markedly modulated the ratio among individual sterols, including an increase in stigmasterol content (26%), a 2-fold increase in sitostanol, induction of cholesterol levels by up to 65%, and a decrease in sitosterol content (35%). Ultimately, elicitation with chitosan, a polysaccharide constituting an element of the fungal cell wall (which is used as a fungal pathogen mimetic), did not significantly increase the biosynthesis and secretion of oleanolic acid saponins in *C. officinalis* hairy roots, but did influence sterol content and the ratio of some compounds. It was assumed that some of the functions of these compounds, including regulation of membrane fluidity and permeability or serving as precursors of brassinosteroids, may be involved in plant defense against fungal attack [59]. Enhancement of sterol and triterpenoid saponin biosynthesis was also observed during elicitation of *C. officinalis* hairy roots with abiotic factors like cadmium and silver ions, ultrasounds, and UV-C radiation [66]. The main phenomenon observed as a response to heavy metal treatment was the stimulation (up to 12-fold) of saponin secretion, which was accompanied by significant changes in sterol composition. Ultrasound stimulated the secretion of saponins (up to 11-fold); however, it exerted diverse influences on the triterpenoid content in hairy root tissue depending on the duration of exposure to the elicitor. UV-C radiation caused a slight increase in the content of both sterols and saponins in hairy root tissue, and stimulated saponin secretion up to 8.5-fold. Thus, the symptoms of the competition between the biosynthetic pathways of sterols and triterpenoids did not appear as evidently in reactions to abiotic stressors as this was reported for biotic elicitors, particularly jasmonic acid [61]. In the study concerning the influence of the other plant hormones, like auxins and cytokinins [62], it was demonstrated that besides one cytokinin, BAP, as described in the previous chapter, all other treatments did not stimulate the biosynthesis of oleanolic acid glycosides, but in some cases increased the sterol levels, e.g., after treatment with kinetin by approx. 17%, particularly influencing the level of isofucosterol (up to 7-fold). It was concluded that, under particular conditions, various natural and synthetic phytohormones applied as elicitors in hairy root cultures may influence varied, often difficult to predict, metabolic pathways [62]. The major results of described studies are briefly summarized in Table 1.

## 9. Discussion

According to the growth-defense trade-off resource hypothesis, one should expect that overlapping metabolic pathways which connect general and specialized metabolism can influence competition and productivity of plant in vitro cultures, including adventitious and hairy roots. Concerning sterols and triterpenoids, the competition mostly appears after squalene cyclization, which has previously been demonstrated in many experiments on wild-type cultures [59,61,62,66]. This effect was often particularly spectacular after treatment with elicitors such as jasmonic acid (JA) or methyl jasmonate (MeJA) [59,61], indicating a regulatory mechanism in plant defense exists. Moreover, this mechanism likely leads to a sharp enhancement of the production of specialized metabolites in response to stress at the cost of general metabolites normally used for basic growth and development. The possibility of genetic modifications for adventitious and hairy roots signifies the importance of tools which attenuate the effects of this redirection, as it was demonstrated in experiments with transgenic cultures (e.g., *P. ginseng*). The most common targets in such studies were 3-hydroxy-3-methylglutaryl CoA reductase (HMGR) [63], farnesyl diphosphate synthase (FPS) [60,64], and squalene synthase (SS) [59,63], while other less frequently studied targets were mevalonate-5-pyrophosphate decarboxylase (MVD) [62] and cycloartenol synthase (CS) [58]. As discussed earlier, these genetic approaches can lead to simultaneous activation of both competing pathways [59,63,67].

Overexpression of single genes (particularly those occurring in early steps in the pathway) may lead to unintended consequences. The experiments performed on hairy roots of *Catharanthus roseus* (L.) G. Don [68] transformed with a hamster gene coding a soluble HMGR, demonstrated an increase in the cytosolic form of HMGR resulting in enhancement of the sterol production rather than increased levels of monoterpene indole alkaloids (including vinblastine, vincristine, ajmalicine and serpentine). This study revealed the interplay between biosynthesis of various specialized metabolites, such as alkaloids and sterols, can be much more complex than just redirection of the metabolic flow. Sterols are components which regulate membrane fluidity and permeability, while also participating in the control of membrane-associated metabolic processes, so elevated sterol concentrations may modify the activity of some enzymes involved in alkaloid biosynthesis, including tonoplast peroxidase that converts ajmalicine to serpentine. Thus, increased sterol accumulation can lead to various unexpected consequences resulting from their regulatory role on membrane property and function.

There have been numerous biotechnological attempts to increase the production of triterpenoids and their saponins [69,70]. In contrast, phytosterols have rarely been treated as the most desirable products that can be obtained from plant in vitro cultures; however, an increasing interest on them has recently surfaced due to their beneficial effects on human health related to hypocholesterolemic capacity and putative antidiabetic, anticancer, and anti-inflammatory properties. Different strategies have been applied to increase the sterol biosynthesis, including elicitation and metabolic engineering [71]. The majority of elicitors (e.g., jasmonates, pectins) caused a decrease in sterol biosynthesis, while the most effective at increasing this process were β-glucan (oligosaccharide derived from the fungal cell wall), (*Z*)-3-hexenol (a fatty acid derivative, “green leaf volatile” compound important in plant damage signalling) and cyclodextrins. However, regardless of the method used for improvement of sterol biosynthesis, the total enhancement of sterol content was not higher more than several times, even in the case of transgenic hairy roots [60]. This phenomenon can be explained by the fact that sterols occur mainly in membranes, so the increase in their production is limited by the site of accumulation. This restriction does not exist with saponins, which can be accumulated in vacuoles or released to the medium, as observed in *C. officinalis* hairy root cultures [61,62].

The present review focused on the interplay between sterols and triterpenoids, and thus did not include the biosynthesis of steroids and steroid saponins in the context of enhancement of their production. The reported studies in this field typically involve diosgenin [72,73], celastrol [74], as well as saponins such as astragalosides [75] and withanolides [76]. As in the case of triterpenoids and triterpenoid saponins, numerous strategies related to redirection of metabolic flux for channelizing the precursor pool toward enhanced steroid or steroid saponin production were attained by deciphering decisive branch points as targets of pathway modulation [77,78,79,80].

Apart from genetic modifications, elicitation remains the simplest and, therefore, the most often applied method to enhance biosynthesis of plant specialized metabolites. Jasmonic acid and its methyl ester (MeJA) are the most commonly applied elicitors in plant in vitro cultures, including adventitious and hairy roots of many plant species, and their efficiency was reported in numerous studies on various compounds [60,61,80,81]. Another plant hormone often applied as a potent elicitor of triterpenoid saponin biosynthesis is salicylic acid (SA) [82]. In contrast, some reports revealed fungi-derived biotic elicitors seem to act on triterpenoid biosynthesis inversely to jasmonates or salicylic acid [61,83]. Regarding the influence of elicitation on biosynthesis pathways of sterol and pentacyclic triterpenoids, one may conclude the mechanism of their regulation does not consist solely of the redirection of the carbon flow at the branch point of squalene cyclization, or limitation in substrate availability. The relationship between sterol and pentacyclic triterpenoids metabolism is much more complicated and may comprise many subsequent feedback loops, e.g., the participation of sterols in membrane microdomains regulating the activity of various enzymes, or glycosyltransferases responsible for saponin formation.

## 10. Concluding Remarks

The competition between the two divergent pathways for sterol and triterpene biosynthesis can hamper the efficient enhancement of the productivity of desired bioactive compounds in plant in vitro cultures, including adventitious and hairy roots of many plant species. This issue can be attenuated or completely suppressed with the properly chosen method of elicitation or genetic modification. Advancements in biotechnological methods of enhancement of biosynthesis of specialized metabolites in plant in vitro cultures throughout the last couple decades have enabled research to combine increased productivity with minimized effects exerted on general metabolism. Thus, recent reports concerning the triterpenoid saponin productivity in various cell, tissue, and organ cultures of plants described in this review often do not contain the analysis of sterol content [84,85,86,87], since the phenomenon of their competition with triterpenoid pathway can be neglected after successful application of a suitable strategy.

Nevertheless, studies on the competition between sterols and triterpenoids were crucial for revealing the pathway of their biosynthesis, understanding the role of particular enzymes, and discovering the possibility of gene regulation. Currently, adventitious and hairy roots of many plant species can be considered not only as an important and efficient tool for controlled production of valuable natural products, but also as suitable experimental models for investigations on regulatory mechanisms of plant development or various aspects of the specific interplay between general and specialized metabolism [88,89].

## Figures and Tables

**Table 1:** The influence of various strategies applied for enhancement of root culture productivity on saponin and sterol contents.

Plant Species/ Experimental Model	Compounds (Triterpenoid Saponins/Sterols)	Strategy Applied to Enhance Saponin Productivity	Effect	Reference
*Panax ginseng* adventitious or hairy roots	ginsenosides (dammarane- and oleanane-type)/ campesterol, sitosterol, stigmasterol	antisense suppression of cycloartenol synthase	ginsenoside content increased by 50–100%, sterol content decreased by 50% in obtained antisense transgenic lines	[58]
		overexpression of squalene synthase	1.6-3-fold increase of ginsenoside content, 2-fold increase of sterol content	[63]
		overexpression of mevalonate-5-pyro- phosphate decarboxylase and farnesyl pyrophosphate synthase	2.4-fold and 4.6-fold increase of ginsenoside and sterol content, respectively, in lines overexpressing farnesyl pyrophosphate synthase; 4.4-fold increase of sterol content (ginsenoside content not changed) in lines overexpressing mevalonate-5-pyrophosphate decarboxylase	[64]
*Bupleurum falcatum* adventitious roots	saikosaponins (oleanane-type)/ campesterol, sitosterol, stigmasterol	elicitation with methyl jasmonate, overexpression of squalene synthase,	elicitation increased saikosaponin content and decreased sterol content in wild-type roots; overexpression of squalene synthase in sense orientation enhanced the level of both saikosaponins and sterols	[59]
*Centella asiatica* hairy roots	centellasaponins, asiaticosides, madecassosides/ campesterol, cholesterol, sitosterol, stigmasterol	overexpression of ginseng farnesyl diphosphate synthase, elicitation with methyl jasmonate	3-fold increase of sterol content (saponin content not affected in transgenic roots, 1.15-fold increase obtained after elicitation with methyl jasmonate	[60]
*Platycodon grandifolium* hairy roots	platycodins (oleanane-type)/ α-spinasterol	overexpression of ginseng 3-hydroxyl-3-methylglutaryl-coenzym A reductase.	up to 2.5-fold and 1.6-fold increase in saponin and sterol content, respectively	[65]
*Calendula officinalis* hairy roots	calendula saponins (oleanane-type)/ cholesterol, campesterol, isofucosterol, sitosterol, stigmasterol	elicitation with jasmonic acid and 6-aminopurine	up to 113-fold increase in saponin release to the medium, 60% decrease in sterol content after elicitation with jasmonic acid; 10-fold increase in saponin release accompanied by 17% decrease in sterol content after elicitation with 6-aminopurine	[61,62]
		elicitation with chitosan	up to 3-fold increase in saponin release to medium, 18% increase in sterol content	[62]
		elicitation with auxins, cytokinins, abiotic elicitors (cadmium and silver ions, UV-irradiation, ultrasound)	up to 12-fold increase in saponin release to the medium, up to 17% increase in sterol content, often accompanied by alterations in sterol profile	[62,66]

## Data Availability

Not applicable.
